# Can the Non-native Salt Marsh Halophyte *Spartina alterniflora* Threaten Native Seagrass (*Zostera japonica*) Habitats? A Case Study in the Yellow River Delta, China

**DOI:** 10.3389/fpls.2021.643425

**Published:** 2021-05-20

**Authors:** Shidong Yue, Yi Zhou, Shaochun Xu, Xiaomei Zhang, Mingjie Liu, Yongliang Qiao, Ruiting Gu, Shuai Xu, Yu Zhang

**Affiliations:** ^1^CAS Key Laboratory of Marine Ecology and Environmental Sciences, Institute of Oceanology, Chinese Academy of Sciences, Qingdao, China; ^2^Laboratory for Marine Ecology and Environmental Science, Qingdao National Laboratory for Marine Science and Technology, Qingdao, China; ^3^Center for Ocean Mega-Science, Chinese Academy of Sciences, Qingdao, China; ^4^CAS Engineering Laboratory for Marine Ranching, Institute of Oceanology, Chinese Academy of Sciences, Qingdao, China; ^5^University of Chinese Academy of Sciences, Beijing, China; ^6^Shandong Province Key Laboratory of Experimental Marine Biology, Qingdao, China; ^7^Qingdao University of Science and Technology, Qingdao, China

**Keywords:** biological invasion, anthropogenic introduction, *Spartina alterniflora*, seagrass, *Zostera japonica*, intertidal zone

## Abstract

Seagrass meadows are critical ecosystems, and they are among the most threatened habitats on the planet. As an anthropogenic biotic invader, *Spartina alterniflora* Loisel. competes with native plants, threatens native ecosystems and coastal aquaculture, and may cause local biodiversity to decline. The distribution area of the exotic species *S. alterniflora* in the Yellow River Delta had been expanding to ca.4,000 ha from 1990 to 2018. In this study, we reported, for the first time, the competitive effects of the exotic plant (*S. alterniflora*) on seagrass (*Zostera japonica* Asch. & Graebn.) by field investigation and a transplant experiment in the Yellow River Delta. Within the first 3 months of the field experiment, *S. alterniflora* had pushed forward 14 m into the *Z. japonica* distribution region. In the study region, the area of *S. alterniflora* in 2019 increased by 516 times compared with its initial area in 2015. Inhibition of *Z. japonica* growth increased with the invasion of *S. alterniflora*. *Z. japonica* had been degrading significantly under the pressure of *S. alterniflora* invasion. *S. alterniflora* propagates sexually via seeds for long distance invasion and asexually by tillers and rhizomes for short distance invasion. Our results describe the invasion pattern of *S. alterniflora* and can be used to develop strategies for prevention and control of *S. alterniflora* invasion.

## Introduction

Biological invasion is a significant component of human-caused global environmental change. Biotic invaders can establish a new range in which they proliferate, spread, and persist to damage the environment (Mack et al., 2000). Animal invaders can cause extinctions of vulnerable native species. For example, the Nile perch (*Lates niloticus*) has contributed to the extinction of more than 200 endemic fish species through predation and competition for food (Lowe et al., 2000). Plant invaders can completely alter the energy budgets and nutrient cycling in a native ecosystem and can greatly diminish the diversity of native species. For example, the water hyacinth (*Eichhornia crassipes*) dramatically reduces biological diversity in aquatic ecosystems through shading and crowding of native aquatic plants (Lowe et al., 2000).

*Spartina alterniflora* is a perennial halophyte that is native to the Atlantic coast of the Americas from Canada to Argentina (Meng et al., 2020). *S. alterniflora* has high tolerance and adaptability to environmental stressors due to fast growth, well-developed below-ground tissues, high salt tolerance, and high reproductive capacity through both asexual and sexual reproduction, making it a good “ecosystem engineer” (Simenstad and Thom, 1995; Qin et al., 1998; Crooks, 2002; Chung et al., 2004; Zhang et al., 2004; Chung, 2006; Huang and Zhang, 2007). Due to these biological traits, *S. alterniflora* has been introduced throughout Europe, North America, Australia, and Asia in efforts to prevent shoreline erosion (Maricle and Lee, 2002). In December of 1979, *S. alterniflora* was introduced into China (Chung, 1993). In 1985, *S. alterniflora* in China covered ∼260 ha (Meng et al., 2020). In 2003, *S. alterniflora* was listed as an invasive species by the State Environment Protection Administration of China. Satellite images showed that the area covered by *S. alterniflora* had reached 34,451 ha in 2007 (Zuo et al., 2012). Today, *S. alterniflora* has expanded to around 50,000 ha of the coastal regions of China (Meng et al., 2020).

As a biotic invader, *S. alterniflora* competes with native plants, threatens native ecosystems and coastal aquaculture, and may cause local biodiversity to decline (Callaway and Josselyn, 1992; Daehler and Strong, 1996; Brusati and Grosholz, 2007; Zuo et al., 2012). The introduction of *S. alterniflora* resulted in a significant decrease of abundance, coverage, and seed and fresh corm output of *Scirpus mariqueter* which is a native species of sedge in Dongtan of Chongming Island (Chen et al., 2004; Wang et al., 2012). Xia et al. (2015) reported that *S. alterniflora* actively accumulated and stored sulfur in its tissues, leading to a high sulfide level in the invaded environment, which was harmful to some native species. Populations of native plants such as *Suaeda glauca* and *Phragmites communis* have decreased rapidly in Yancheng National Nature Reserve due to expansion of alien *S. alterniflora*, and the habitat changes are believed to be at least partially responsible for dramatic declines of wintering red-crowned crane populations (Liu C. et al., 2013). Ge et al. (2012) reported that the biodiversity of macrobenthic communities increased in the initial stage of *S. alterniflora* invasion and then decreased in the middle and final stage of invasion in Wenzhou Bay, China.

Seagrasses provide habitat, food, and nurseries for a variety of marine organisms, attenuate currents and waves, alter nutrient cycling and food web structures, and stabilize sediments (Costanza et al., 1997; Jackson et al., 2001; Barbier et al., 2011; Liu X.J. et al., 2013; Nordlund et al., 2018; Unsworth et al., 2019). Seagrass meadows are critical ecosystems, and they are among the most threatened habitats on the planet. Waycott et al. (2009) reported that 29% of the known areal extent of seagrass meadows has disappeared since seagrass areas were initially recorded in 1879. Human disturbances such as eutrophication and habitat loss due to dredging, anchoring, and coastal construction play a key role in the loss of seagrasses (Orth et al., 2006; Ralph et al., 2006; Salinas et al., 2020).

After *S. alterniflora* was first introduced to the Yellow River Delta in 1990, it grew and spread rapidly. By 2018, its distribution region had expanded to 4005.89 ha, which was 2557 times as its initial colonization area (Ren et al., 2019). Zhang X.M. et al. (2019) reported that the seagrass *Zostera japonica* Asch. & Graebn. on both sides of the mouth of the Yellow River covered about 1031.8 ha. *Z. japonica* is an intertidal seagrass species that is native to the Western Pacific Ocean from Russia to Vietnam (Miki, 1933). This species is widely threatened by climate change and human activity in its native range in Korea, Japan, and China (Lee et al., 2004; Abe et al., 2009; Hodoki et al., 2013; Zhang et al., 2014, 2015, 2020a,b; Lin et al., 2018; Yue et al., 2020). The niche of *S. alterniflora* overlaps with that of *Z. japonica* on low tidal beaches. However, competition between *S. alterniflora* and *Z. japonica* has rarely been studied experimentally in the field.

In May 2015, we found an initial invasion of *S. alterniflora* (ca.100 m^2^) at *Z. japonica* meadows in our study region, and *S. alterniflora* had been occurring in small patches surrounded by *Z. japonica* meadows. In this study, we investigated the competitive effects of *S. alterniflora* on *Z. japonica* in the study region to assess the possible consequences of the introduced *S. alterniflora* on the native *Z. japonica* communities in the Yellow River Delta. We document the invasion pattern of *S. alterniflora* by *in situ* investigation and our work can be used to develop strategies for prevention and control of *S. alterniflora* invasion.

## Materials and Methods

### Study Sites

**FIGURE 1:**
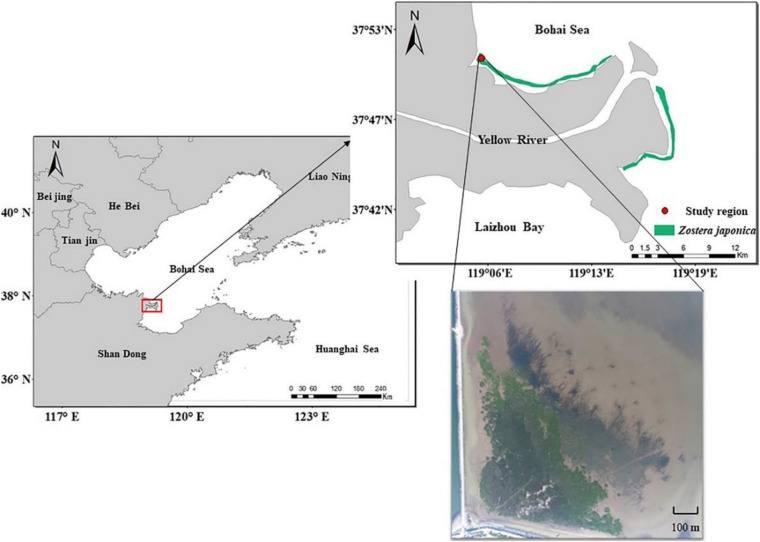
Study region in the Yellow River Delta.

The study region (37° 51′ 7″ N, 119° 5′ 47″ E) is located in the Yellow River Delta, Shandong Province, China (Figure 1). The climate is temperate monsoonal, with an average annual precipitation of 560 mm and average annual temperature of 12.9°C (Han et al., 2018). The tides are irregular semidiurnal, with an average tidal amplitude of 1.1–1.5 m (Hu and Cao, 2003). The broad mudflat of the Yellow River Delta displays distinct vegetation zones, producing a unique wetland landscape extending from sea to land. *Z. japonica* meadows occur in the sea, with *S. alterniflora*, *Suaeda salsa*, *Tamarix chinensis*, and reed marsh occurring in the landward direction (Zhang X.M. et al., 2019). Based on the vegetation differences, the study region can be divided into the *S. alterniflora* distribution region, the ecotone (*S. alterniflora* and *Z. japonica*) and the *Z. japonica* distribution region.

### Distribution of *Spartina alterniflora* and *Zostera japonica* in the Study Region

In May 2015, we used mobile phone to record the distribution of *S. alterniflora* in the study region. In October 2016 and July 2019, the total distribution region of *S. alterniflora* was photographed by aerial vehicle (DJI Phantom 3 Advanced) during low tide. Then we estimated the distribution area of *S. alterniflora* by using road as a reference.

In July 2019, during peak biomass, the range of *Z. japonica* meadows was examined by walking during low tide or by rowing during mid to high tide, using GPS to record accessible boundary points.

To estimate the sediment accretion due to the invasion of *S. alterniflora* in the study region, we recorded the corresponding tide level from the same place (37° 51′ 6″ N, 119° 5′ 53″ E) exposed to the sea surface in May 2015 and May 2019. The tidal regime for the study region was provided by the National Marine Data Information Center of China.

### Environmental Parameters

Four sediment cores (diameter = 10.5 cm, depth = 12 cm) were collected from each region to measure grain size distribution using laser diffraction analysis (Short and Coles, 2001). These sediment cores were also used to determine the concentration of sediment organic matter (OM, in %DW), which was calculated as the fractional weight loss of dry sediment in the samples after combustion at 550°C for 4 h (Heiri et al., 2001). To compare the sulfide content in sediment in the *S. alterniflora* and the *Z. japonica* distribution regions, four sediments cores (diameter = 10.5 cm, depth = 12 cm) were collected from the two regions and measured in the laboratory by using the iodometric method (National marine environmental monitoring center [NMEMC], 2007).

### Field Investigation

Three parallel transect lines running perpendicular to the shoreline were marked for sampling in the study region. All transects began in the *S. alterniflora* distribution region and ended in the *Z. japonica* distribution region. Eleven sampling points were marked at an interval of 7 m along each transect line. Sampling was conducted in April, July, and October, 2019. One sediment core (diameter = 10.5 cm, depth = 12 cm) was haphazardly collected within 1 m of each sampling point along each transect.

All samples were sieved (2 mm) with seawater *in situ* to remove most of the sediment and then cleaned using tap water in the laboratory. For each sample, total number of shoots was counted to provide shoot density (shoots ⋅ m^–2^). The height (cm) of 15 shoots was measured. All shoots were dried to a constant weight at 60°C to estimate total shoot biomass (g DW ⋅ m^–2^). We also counted the density (shoots ⋅ m^–2^), height (cm), and biomass (g WW ⋅ m^–2^) of seedlings, which were only observed in April, 2019. All of these biological parameters were measured separately for *S. alterniflora* and *Z. japonica*.

### Transplant Experiment

We designated three transplant sites (S1, S2, and S3) for the experiment in which we transplanted *Z. japonica*. S1 was located in the *S. alterniflora* distribution region, which represented the completed stage of invasion. S2 was located at the edge of the *S. alterniflora* distribution region, which represented the intermediate stage of invasion. S3 was located in the ecotone, which represented the initial stage of invasion.

All transplant experiments began in July, 2019. For each transplant site, four transplant plots (1 m × 1 m) were designed parallelly to the coast with an interval of 2 m. We assumed that *Z. japonica* might not grow at S1, thus we added another three transplant plots (1 m × 1 m) at S1 to ensure adequate monitoring data. In each plot, all *S. alterniflora* were removed using a sickle and hoe. Sod consisting of *Z. japonic*a and sediment was collected from the center of the *Z. japonica* distribution region using tools for pushing the plant material horizontally below the rhizome layer. Then, these sods were transplanted into the pre-dug plots from which *S. alterniflora* were removed.

After transplantation, seven sediment cores (diameter = 10.5 cm, depth = 12 cm) were collected semimonthly at S1, and four sediment cores (diameter = 10.5 cm, depth = 12 cm) were collected semimonthly at S2 and S3. All cores were sieved (2 mm) with seawater *in situ* to remove most of the sediment, and the plant materials then were taken back to the laboratory. For each sample, plant material was washed using tap water and divided into above-ground (shoots: sheath and leaves) and below-ground (rhizomes and roots) parts. The shoot height (cm) was measured and the numbers of vegetative and flowering shoots were counted to provide shoot density (shoots ⋅ m^–2^). Total tissues were dried to a constant weight at 60°C to estimate total above-ground and below-ground biomass (g DW ⋅ m^–2^).

### Statistical Analysis

For the field investigation data, we used a one-way analysis of variance (ANOVA) to test the significance of differences in the total shoot density, biomass of *S. alterniflora*, and *Z. japonica* among time and sampling points and to test for significance of differences in seedling density and biomass among the 11 sampling points along the transect line. The significance of differences in grain sizes and OM among three regions was also tested by using a one-way ANOVA. An independent-samples *t*-test was used to test the significance of differences in the sulfide content in sediment among *S. alterniflora* and *Z. japonica* distribution regions. For the transplant experiment data, we used one-way ANOVA or independent-samples *t*-test to test the significance of differences in the reproductive shoot density, vegetative shoot density, total shoot density, below-ground biomass, above-ground biomass, total biomass, vegetative shoot height, and reproductive shoot height among time points and transplant sites. When the data did not satisfy the homogeneity of variance requirement, a Kruskal-Wallis Test was used to test the significance of differences. Multiple comparisons were performed using the Duncan method, and the level of significance was set at *p* < 0.05. Statistical analyses were conducted using SPSS 17.0. All values are reported as mean ± SD.

## Results

### Distribution of *Spartina alterniflora* and *Zostera japonica* in the Study Region

**FIGURE 2:**
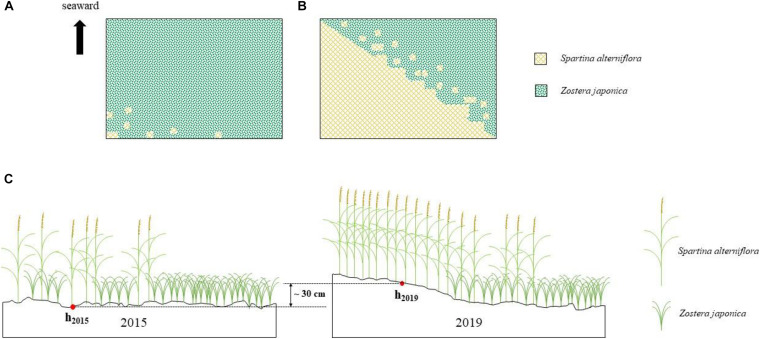
*Spartina alterniflora* invasion of *Zostera japonica* meadows [**(A)** 2015; **(B)** 2019] and the sediment accretion in the study region **(C)**.

In 2015, the area of *S. alterniflora* was ca.100 m^2^. In 2016, it had extended over ca. 1.25 ha. By 2019, the distribution region had expanded to ca. 5.16 ha (Figures 2A,B; Supplementary Figure 1).

The distance between the shore at the study region to the lower limits of *Z. japonica* meadows was 537 m, while the distance between the upper and lower limits of *Z. japonica* meadows was only 165 m in July 2019.

The place was exposed to the sea surface at the tidal water level of 50 cm in May 2015, while it was exposed to the sea surface at the tidal water level of 80 cm in May 2019. It shows that approximately 30 cm sediment was accumulated owing to the invasion of *S. alterniflora* in the study region (Figure 2C).

### Environmental Parameters

**FIGURE 3:**
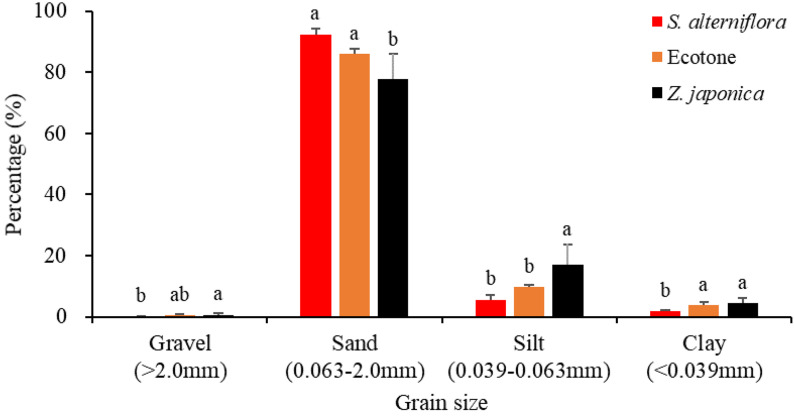
Sediment grain sizes at the three regions. Values are mean ± SD. Different letters indicate significant difference between different regions at same size of grain.

The sediment particulate size composition in all the regions was mainly sand (Figure 3), while the percentage of sand at *S. alterniflora* distribution region was greater than at *Z. japonica* distribution region [*F*_(__2_,_9__)_ = 8.887, *p* < 0.05]. There was no significant difference in OM among region [Table 1; *F*_(__2_,_9__)_ = 0.646, *p* > 0.05]. The sulfide content of sediment in *S. alterniflora* distribution region (237.50 ± 88.88 mg ⋅ kg^–1^) was greater than in *Z. japonica* distribution region (156.25 ± 58.55 mg ⋅ kg^–1^).

**TABLE 1:** The concentration of sediment organic matter and sulfide content at the three regions.

Region	OM (%)	Sulfide (mg ⋅ kg^–1^)
*Spartina alterniflora*	2.93 ± 0.30	237.50 ± 88.88
Ecotone	2.72 ± 0.38	–
*Zostera japonica*	2.75 ± 0.08	156.25 ± 58.55

*Values are mean ± SD.*

### Competition Between *Spartina alterniflora* and *Zostera japonica*

**FIGURE 4:**
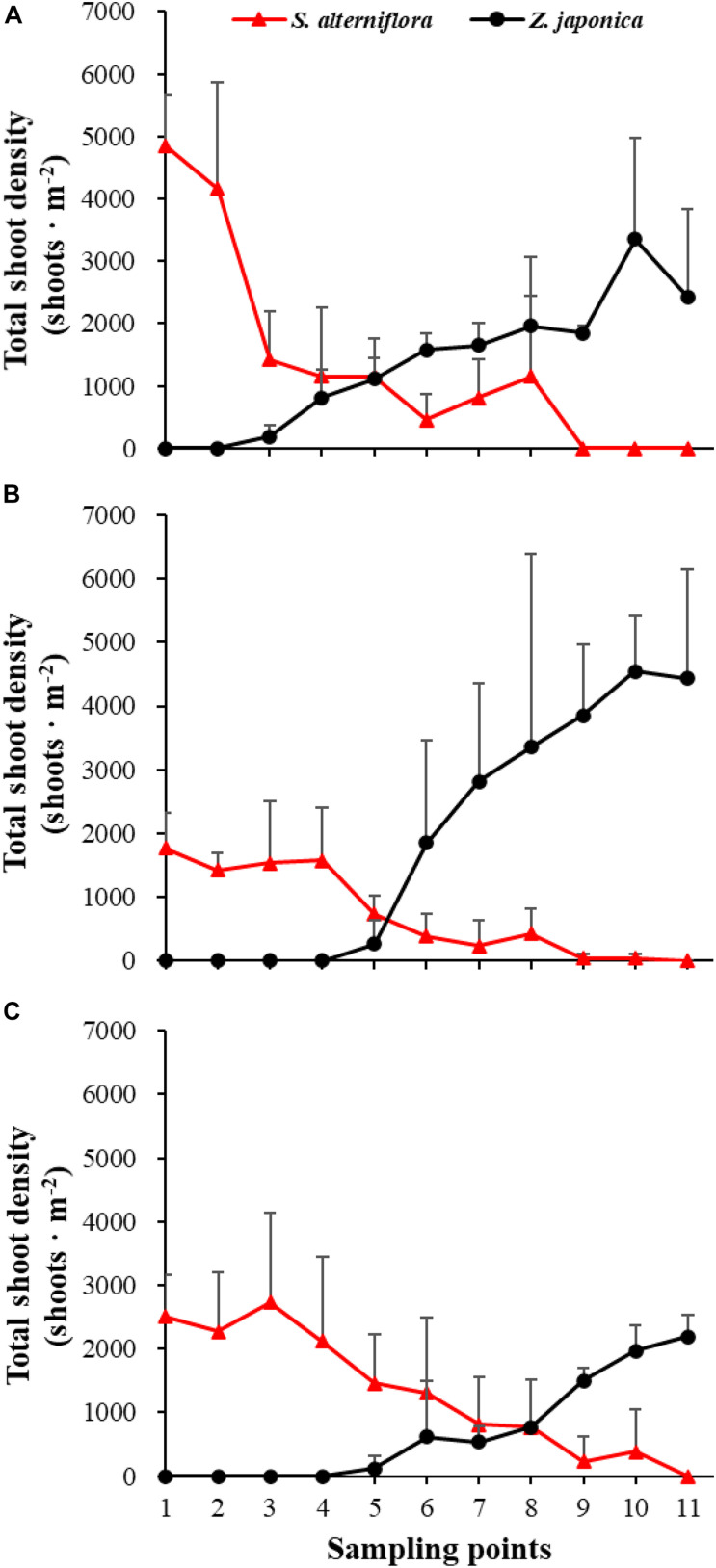
Total shoot density of *Spartina alterniflora* and *Zostera japonica* at the 11 sampling points in April **(A)**, July **(B)**, and October **(C)** 2019. All *S. alterniflora* shoots collected at sampling points 9 and 10 in July 2019 were grown from dormant seeds that germinated in May 2019. Values are mean ± SD.

**FIGURE 5:**
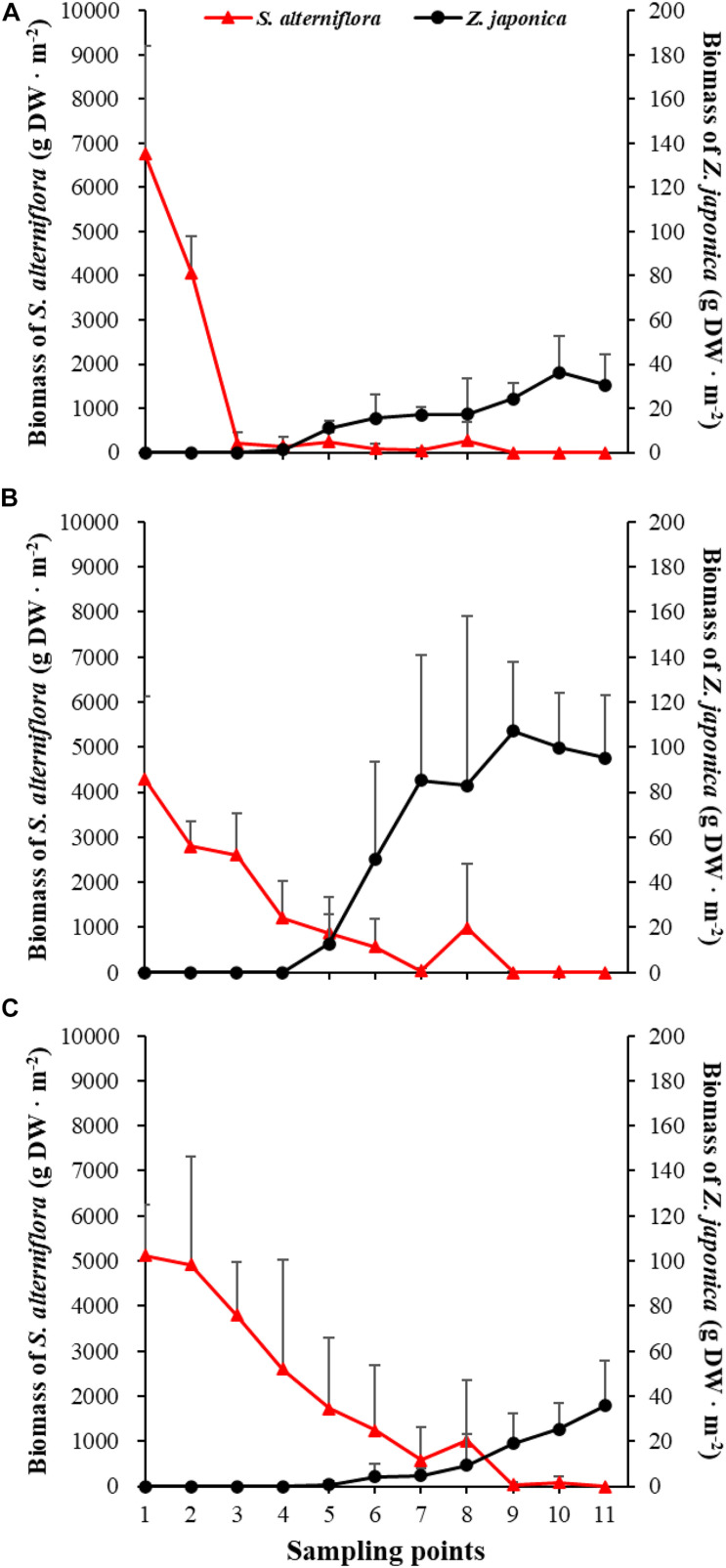
Biomass of *Spartina alterniflora* and *Zostera japonica* at the 11 sampling points in April **(A)**, July **(B)**, and October **(C)** 2019. Values are mean ± SD.

There were significant differences among sampling points in total shoot density, biomass of *S. alterniflora*, and *Z. japonica* in each sampling time (*p* < 0.05). The total shoot density of *S. alterniflora* and *Z. japonica* decreased and increased with moving seaward, respectively (Figure 4). Biomass showed the same trend as shoot density (Figure 5). In April, the biomass of *S. alterniflora* at points 5 and 8 was higher than at the adjacent points (Figure 5A). In July and October, the biomass of *S. alterniflora* at point 8 was still higher than that of the adjacent points (Figures 5B,C).

At point 1, the total shoot density of *S. alterniflora* in April (4,852.90 ± 808.82 shoots ⋅ m^–2^) was higher than that in October (2,503.48 ± 657.02 shoots ⋅ m^–2^) and July (1771.69 ± 546.05 shoots ⋅ m^–2^) [*F*_(__2_,_6__)_ = 16.852, *p* < 0.05]. At point 3, the biomass of *S. alterniflora* in April (1,153.14 ± 1,280.93 g DW ⋅ m^–2^) was lower than that in July (2,612.86 ± 903.89 g DW ⋅ m^–2^) and October (3,799.51 ± 1,176.13 g DW ⋅ m^–2^) [*F*_(__2_,_6__)_ = 13.330, *p* < 0.05]. At points 4 and 5, the total shoot density of *Z. japonica* decreased with sampling time [*F*_(__2_,_6__)_ = 9.188, *p* < 0.05; *F*_(__2_,_6__)_ = 9.046, *p* < 0.05, respectively]. At point 9, the total shoot density of *Z. japonica* was highest in July (3,851.51 ± 1110.28 shoots ⋅ m^–2^), followed by April (1848.72 ± 115.55 shoots ⋅ m^–2^) and October (1,502.09 ± 200.13 shoots ⋅ m^–2^) [*F*_(__2_,_6__)_ = 11.256, *p* < 0.05]. At points 7, 9, 10, and 11, the biomass of *Z. japonica* was highest in July, followed by April and October (*p* < 0.05).

**FIGURE 6:**
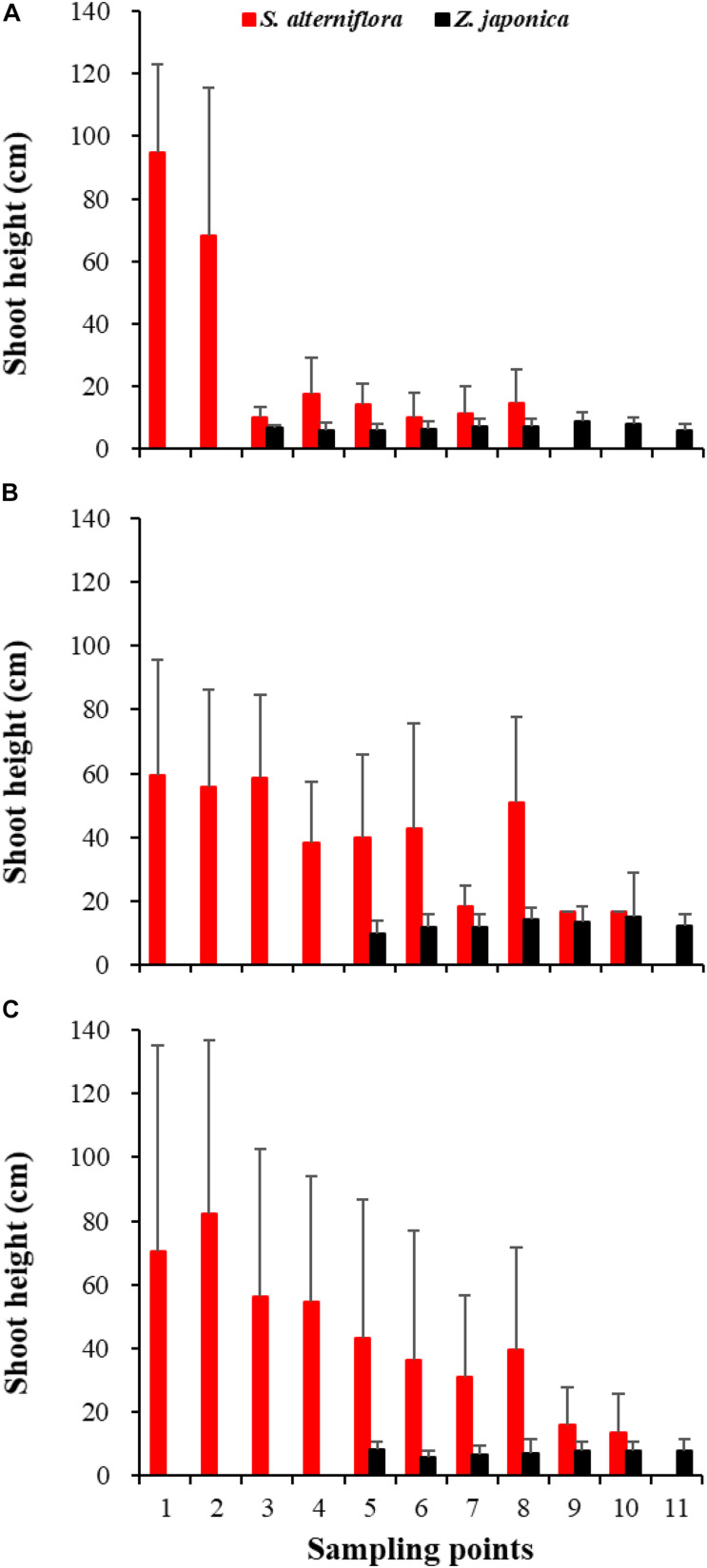
Shoot height of *Spartina alterniflora* and *Zostera japonica* at the 11 sampling points in April **(A)**, July **(B)**, and October **(C)** 2019. Values are mean ± SD.

In April, *Z. japonica* was observed from point 3 to point 11 and *S. alterniflora* was observed from point 1 to point 8 (Figure 6A). In July and October, *Z. japonica* was no longer observed at points 3 and 4, and *S. alterniflora* was observed at points 9 and 10 (Figures 6B,C). *S. alterniflora* shoots collected at sampling points 9 and 10 in July 2019 had small shoot height (<20 cm), biomass (<10 g WW ⋅ m^–2^), and density (<50 shoots ⋅ m^–2^); these shoots could be easily uprooted and were obviously grown from dormant seeds that germinated in May 2019. This finding shows that *S. alterniflora* had pushed forward 14 m into the *Z. japonica* distribution region.

**FIGURE 7:**
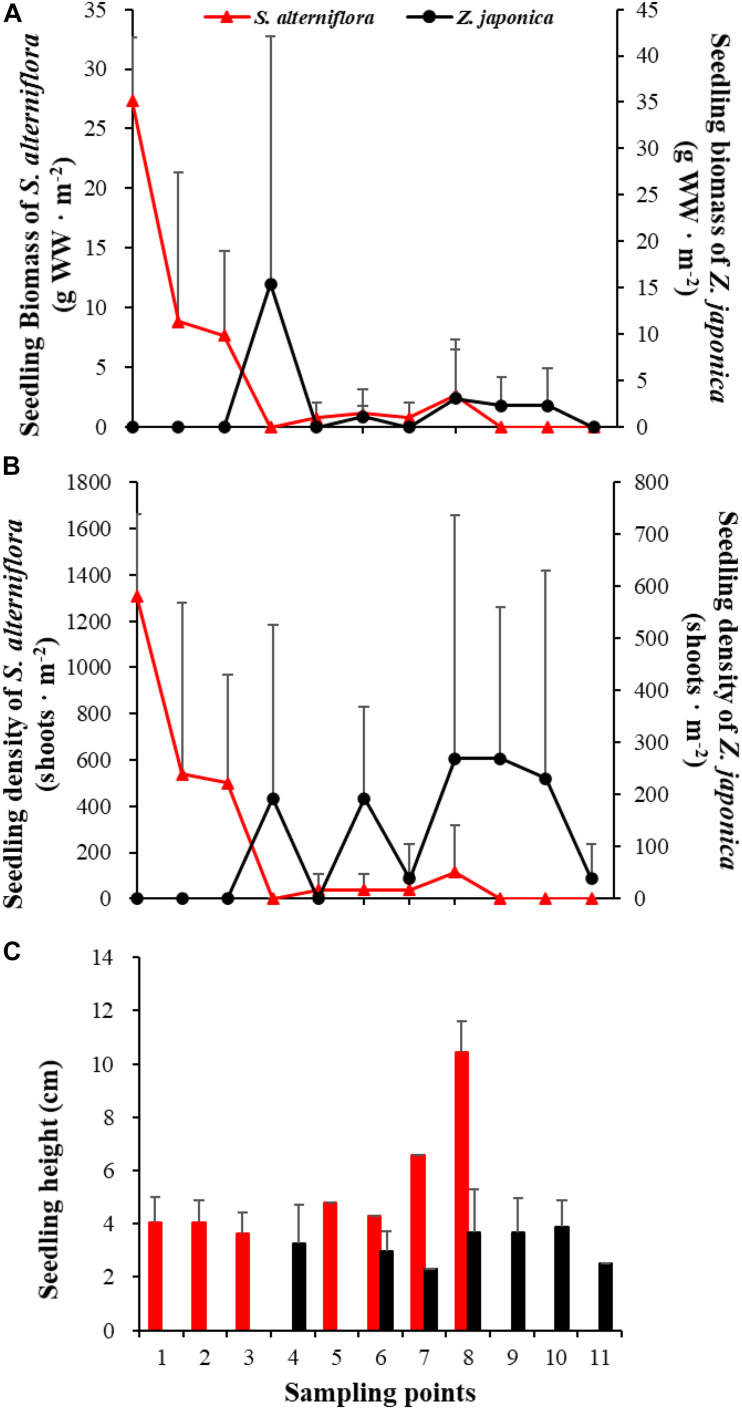
Seedling biomass **(A)**, density **(B)**, and height **(C)** of *Spartina alterniflora* and *Zostera japonica* at the 11 sampling points in April 2019. Values are mean ± SD.

In April, seedlings of *S. alterniflora* and *Z. japonica* were found in the samples. The seedling density and biomass of *S. alterniflora* differed significantly among sampling points [*F*_(__10_,_22__)_ = 5.809, *p* < 0.05; *F*_(__10_,_22__)_ = 8.459, *p* < 0.05, respectively]. The seedling biomass of *S. alterniflora* and *Z. japonica* decreased and increased with moving seaward, respectively (Figure 7A). Seedling density showed the same as biomass (Figure 7B). At point 8, the seedling density (115.55 ± 200.13 shoots ⋅ m^–2^) and biomass (2.70 ± 4.67 g WW ⋅ m^–2^) of *S. alterniflora* were higher than at adjacent points (Figure 7). However, seedling density and biomass of *Z. japonica* did not differ significantly among sampling points [*F*_(__10_,_22__)_ = 0.741, *p* > 0.05; *F*_(__10_,_22__)_ = 0.883, *p* > 0.05, respectively].

### Effects of Different *Spartina alterniflora* Invasion Stage on *Zostera japonica*

**FIGURE 8:**
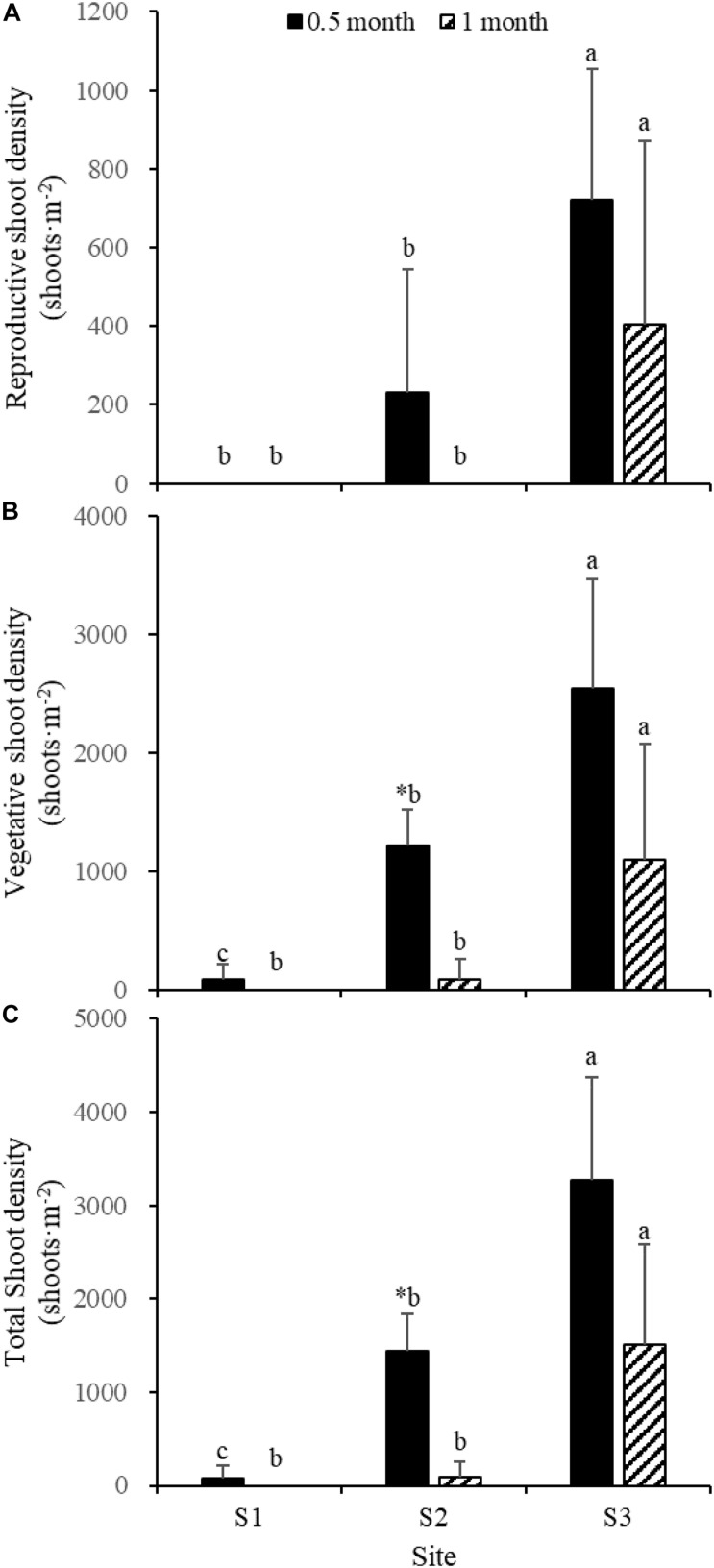
Reproductive shoot density **(A)**, vegetative shoot density **(B)**, and total shoot density **(C)** of *Zostera japonica* in the three transplant sites at 0.5 and 1 month after transplantation. Values are mean ± SD. ^∗^Indicates significant difference between different time points in the same transplant site. Different letters indicate significant difference between different transplant sites at the same time point.

Fifteen days after transplantation, the reproductive shoot density, vegetative shoot density, and total shoot density of *Z. japonica* at S1 were lower than those at S2 and S3 [Figure 8; Chi-square_(__2__)_ = 11.343, *p* < 0.05; Chi-square_(__2__)_ = 11.809, *p* < 0.05; *F*_(__2_,_12__)_ = 36.916, *p* < 0.05, respectively]. The above-ground biomass, total biomass, vegetative shoot height, and reproductive shoot height at S1 were lower than those at S2 and S3 [Figures 8, 10; *F*_(__2_,_12__)_ = 20.697, *p* < 0.05; *F*_(__2_,_12__)_ = 6.973, *p* < 0.05; *F*_(__2_,_12__)_ = 8.073, *p* < 0.05; Chi-square_(__2__)_ = 8.480, *p* < 0.05, respectively]. One month after transplantation, all *Z. japonica* at S1 had died. The total shoot density and biomass at S2 were lower than those at S3 [Figures 7, 8; Chi-square_(__2__)_ = 7.659, *p* < 0.05; Chi-square_(__2__)_ = 8.199, *p* < 0.05, respectively].

**FIGURE 9:**
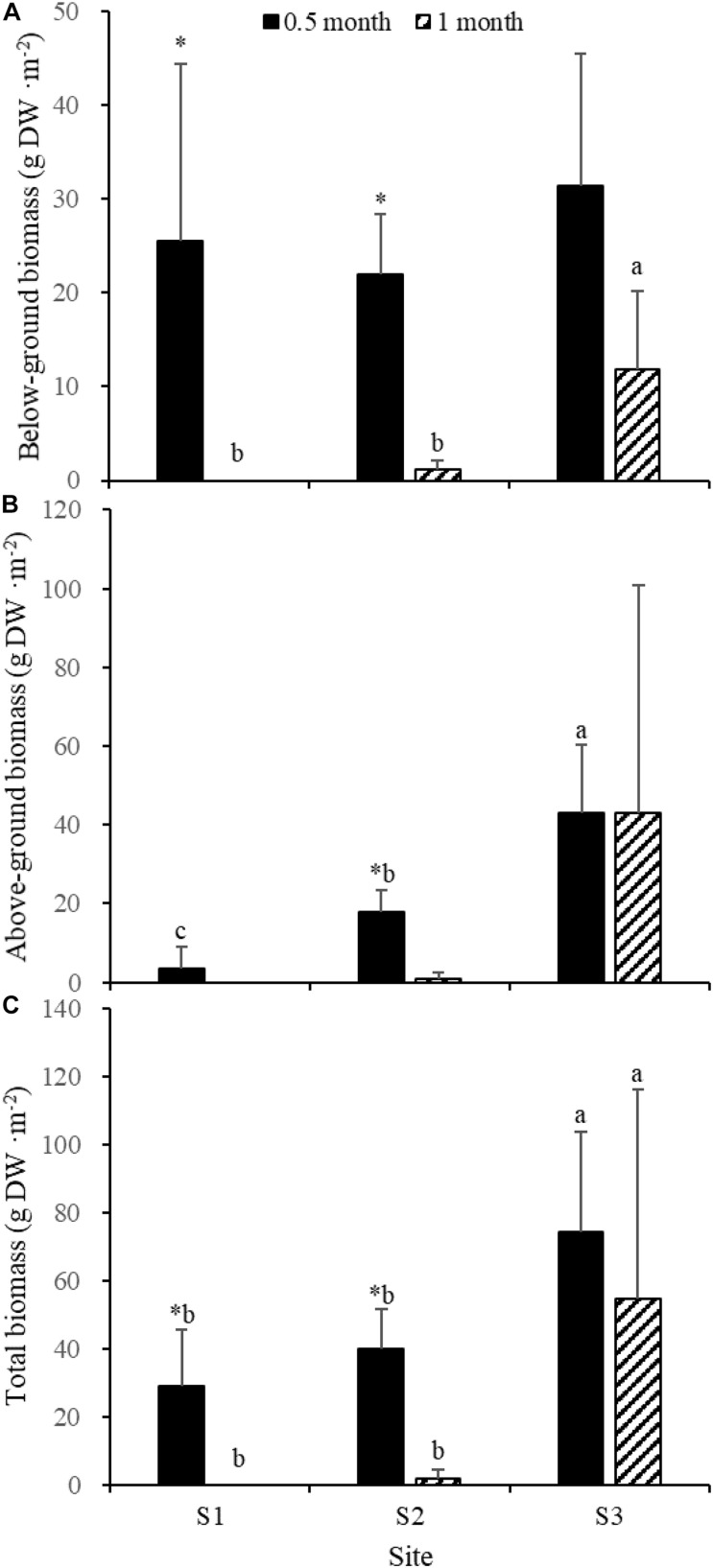
Below-ground biomass **(A)**, above-ground biomass **(B)**, and total biomass **(C)** of *Zostera japonica* in the three transplant sites at 0.5 and 1 month after transplantation. Values are mean ± SD. ^∗^Indicates significant difference between time points in the same transplant site. Different letters indicate significant difference between different transplant sites at the same time point.

**FIGURE 10:**
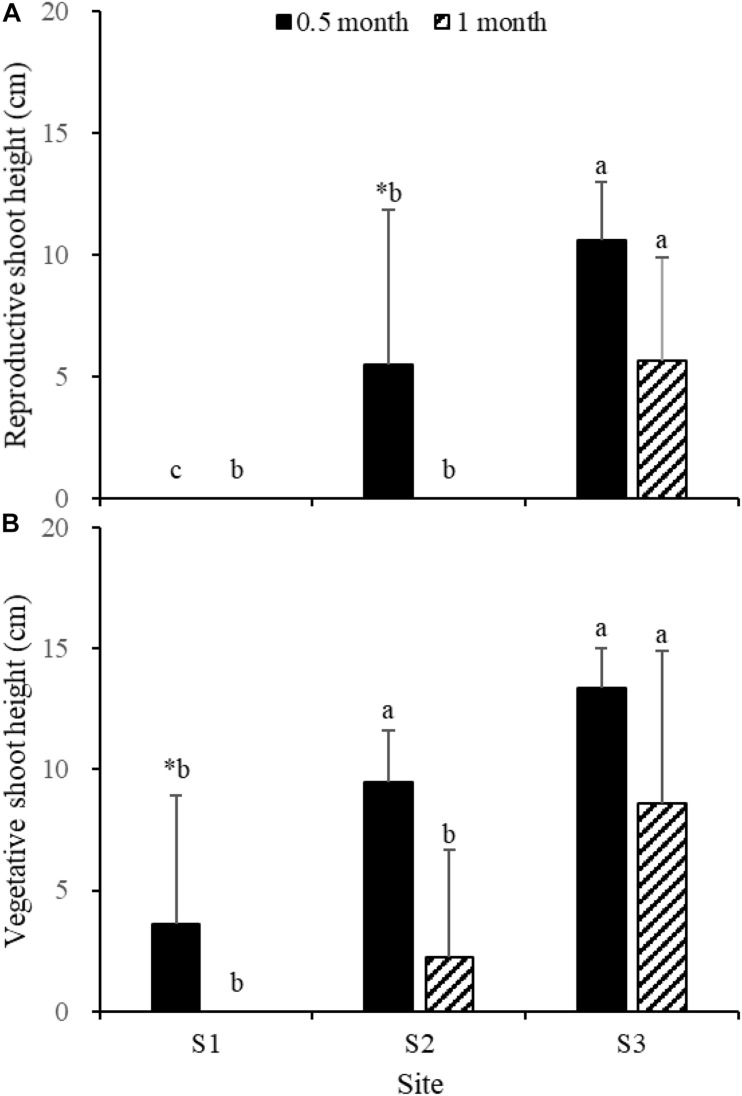
Reproductive shoot height **(A)** and vegetative shoot height **(B)** of *Zostera japonica* in the three transplant sites at 0.5 and 1 month after transplantation. Values are mean ± SD. ^∗^Indicates significant difference between time points in the same transplant site. Different letters indicate significant difference between different transplant sites at the same time point.

No significant differences among time points for total shoot density and biomass at S3 were detected [Figures 8, 9; *t*_(__6__)_ = 2.289, *p* > 0.05; *t*_(__6__)_ = 0.586, *p* > 0.05, respectively]. The vegetative shoot height and reproductive shoot height at S3 decreased over time (Figure 10).

## Discussion

In this study, we reported, for the first time, the competitive effects of the exotic plant (*S. alterniflora*) on seagrass (*Z. japonica*) by field investigation and a transplant experiment in the Yellow River Delta. Our results indicated that the seagrass meadow had been degrading significantly under the pressure of *S. alterniflora* invasion. Our results document the invasion pattern of *S. alterniflora* and can be used to develop strategies for prevention and control of *S. alterniflora* invasion.

### Distribution of *Spartina alterniflora* and *Zostera japonica* in the Study Region

By 2019, *S. alterniflora* covered ca. 5.16 ha, which was 516 times greater than its initial area in 2015. In Jiangsu Province, the total planting area of *S. alterniflora* was 278.93 ha along the north coast of Sheyang in 1982–1992, but it rapidly spread and increased to 1160.69 ha by July 1, 1994 (Zuo et al., 2012). Ren et al. (2019) used remote sensing technology to monitor distributional changes of *S. alterniflora* in the Yellow River Delta. They reported that *S. alterniflora* was first identified in Landsat 5 images in 2008 on the north bank with an initial area of 0.72 ha, whereas it was first identified on the south bank in 2011 with an initial area of 1.61 ha. Since 2011, *S. alterniflora* has rapidly colonized the modern Yellow River Delta. Chen et al. (2020) reported that Fast Super-Resolution Convolutional Neural Networks (FSRCNN) were very effective at discerning and estimating the size of small *S. alterniflora* patches (<50 m^2^). They found that the total patch area of *S. alterniflora* at one site increased 13 times from 11.54 ha to 157.42 ha since 2012.

The distance between the upper and lower limits of *Z. japonica* meadows was greater than 550 m in 2015 (Zhang X.M. et al., 2019), while the distance we observed was only 165 m in July 2019. The result means *Z. japonica* was seriously degraded and had not moving seaward under the pressure of *S. alterniflora* invasion.

In the Yellow River Delta, the invasion of *S. alterniflora* is a common phenomenon at the *Z. japonica* meadows. At another site, a limited initial invasion of *S. alterniflora* (ca.30 m^2^) at *Z. japonica* meadows was observed in August, 2015; however, after 5 years, large areas of this site were also invaded by *S. alterniflora* (Supplementary Figure 2).

### Invasion Pattern of *Spartina alterniflora*

In our field investigation, *S. alterniflora* pushed forward 14 m into the *Z. japonica* distribution region between April and July 2019, which illustrates its powerful invasion ability. Liu et al.(2020a,2020b) reported that the height, density, above-ground biomass, and reproductive ability of *S. alterniflora* in the Chinese range were greater than those in the native range.

Seedlings of *S. alterniflora* are smaller than adult shoots and can reach sexual maturity within 3–4 months (Meng et al., 2020). In this study, *S. alterniflora* seedlings were recorded in spring, while seed maturation occurred in Autumn, indicating that seeds did not germinate immediately after maturity. *S. alterniflora* propagates sexually via seeds and asexually by tillers and rhizomes. *S. alterniflora* shoots collected at sampling points 9 and 10 in July 2019 could be easily uprooted and were very small; these shoots were obviously grown from dormant seeds that germinated in May 2019.

*S. alterniflora* has aerenchyma, making it adaptable to the anoxic environment (Meng et al., 2020). Therefore, seedlings could survival in the *Z. japonica* distribution region that was characterized by relatively deep seawater. We observed multiple peaks of biomass of *S. alterniflora* as the sampling point number increased, which represented the irregularly shaped patches of *S. alterniflora*. In the initial stages of *S. alterniflora* invasion, sparse patches arising from germinating seeds are distributed on the tidal flats (Ma et al., 2019).

*S. alterniflora* has a high tolerance and adaptability to environmental stressors (including lower temperature). During winter, the above-ground tissues of *S. alterniflora* turn yellow but keep standing (Supplementary Figure 3). Therefore, *S. alterniflora* would not die off at sampling points 9 and 10 throughout the winter. In addition, we investigated the growth of *S. alterniflora* at sampling points 9 and 10 in June, 2020; the results showed that the shoot height was greater than 25 cm, and the shoot density was greater than 250 shoots ⋅ m^–2^. These values were greater than those in October 2019 (except the shoot density at point 10). These data indicated that *S. alterniflora* at sampling points 9 and 10 had spread locally by clonal growth in 2020.

We found that the biomass of *Z. japonica* increased in July and decreased in October within the *Z. japonica* distribution region. However, the biomass of *Z. japonica* decreased over time in the front part of the ecotone. Zhang X.M. et al. (2019) previously described the seasonal changes in *Z. japonica* in the Yellow River Delta. They reported that growth increased in June and July, peaked in August, and dramatically declined by October. The higher height of *S. alterniflora* shielded the sunlight originally used by *Z. japonica* and thus inhibited growth of the seagrass. The biomass of *S. alterniflora* in October was higher than that in July, which indicated that the growth ability of *S. alterniflora* was greater than that of *Z. japonica*.

Over the past century, the coastal erosion of many deltas was triggered by the decrease of sediment loads of many rivers owing to damming and irrigation as well as improved land-use practices, such as the Yellow River Delta (Milliman, 1997; Wang et al., 2007; Syvitski et al., 2009; Bi et al., 2014; Jiang et al., 2017). Waves impacting the shoreline can suspend sediment while currents can transport these materials elsewhere, causing erosion (Fan et al., 2018). Higher growth rates of *S. alterniflora* promote greater standing biomass, which in turn slows water velocity on the marsh platform, lowers wave height, reduces erosion, and enhances mineral sediment deposition (Kirwan and Megonigal, 2013). It is suggested that the invasion of *S. alterniflora* increased the accumulation of sediment. Gao et al. (2014) reported that *S. alterniflora* can enhance the settling flux of suspended sediment and the deposition rate on the tidal flats by reducing the near-bed shear stress associated with tidal currents. Sediment accretion may increase the ecological niche of *S. alterniflora*, which is one potential explanation for the successful invasion of *S. alterniflora* into *Z. japonica* meadows.

**FIGURE 11:**
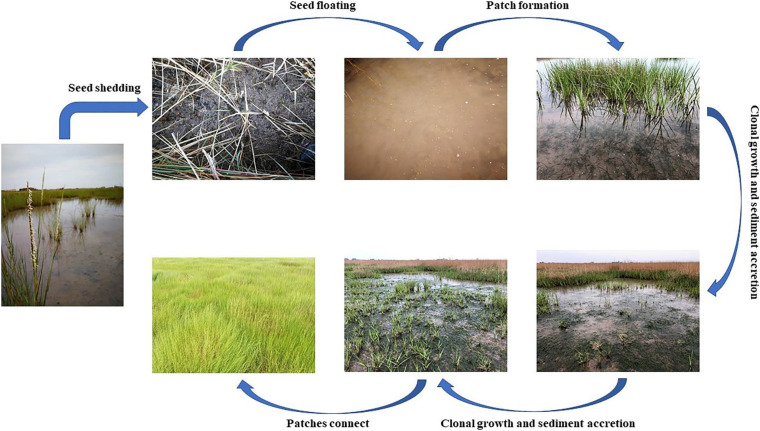
The invasion pattern of *Spartina alterniflora* in *Zostera japonica* meadows.

*S. alterniflora* propagates sexually via seeds for long distance invasion and asexually by tillers and rhizomes for short distance invasion (Figure 11). The seeds fall off the plant after maturing and float with the tide into the seagrass bed, where sparse patches of the invader arise from germinating seeds. Subsequently, the deposition rate increases gradually as the density of clonal ramets increases, and over time the growth of *Z. japonica* is inhibited. Ultimately, patches of *S. alterniflora* connect and replace the seagrass bed community through vegetative reproduction.

### Ecological Effects of *Spartina alterniflora* Invasion

Results of the transplant experiment showed that inhibition of *Z. japonica* growth increased with the invasion stage of *S. alterniflora*. The ecological functions of *Z. japonica* meadows may be reduced by invasion of *S. alterniflora*. Zhang Y. et al. (2019) reported that the invasion of *S. alterniflora* into the Chinese coastal wetlands caused profound biotic homogenization of soil communities across latitudes. In the Chongming wetland of Shanghai, the species and number of birds in the *S. alterniflora*-vegetated community were much lower than in native reed communities (Meng et al., 2020). The Yellow River Delta National Nature Reserve is an important transit point, habitat, and breeding ground for bird migration. A high-density *S. alterniflora* population can act as an “isolation belt” between birds and their food resources, which may decrease bird diversity.

Significant quantities of carbon can be sequestered by, stored in, and released from seagrass meadows (Prentice et al., 2020). Salinas et al. (2020) reported that the loss of seagrass due to human and natural disturbances may have caused the release of 11–21 Tg CO_2__–eq_ in Australia since the 1950s. Yang et al. (2013) reported that soil organic carbon was significantly increased in the upper 0–30 cm soil layer after *S. alterniflora* invaded the *S. salsa* and *Cyperus malaccensis* communities. Wang et al. (2019) found that the soil total C content was 13.3 ± 0.3 t ⋅ ha^–1^ in the native mangrove regions, while the soil total C content was 10.9 ± 0.3 t ⋅ ha^–1^ in the invasive *S. alterniflora* regions. Thus, the carbon buried in *Z. japonica* meadows may change after *S. alterniflora* invasion. Measuring soil organic carbon (SOC,%) by organic matter (OM,%) analyzer can significantly reduce cost per sample analysis over the long-term (Howard et al., 2014). Craft et al. (1991) reported the relationships between OM (%) and SOC (%) in tidal salt marsh [SOC = 0.40 × OM + 0.0025 (OM)^2^, *r*^2^ = 0.99]. Also, Fourqurean et al. (2012) reported the relationships between OM (%) and SOC (%) in seagrass meadows (SOC = 0.43 × OM – 0.33, *r*^2^ = 0.96). Therefore, we used the first equation to estimate the SOC content in the *S. alterniflora* distribution region and the ecotone. The SOC content in the *Z. japonica* distribution region was estimated by the second equation. The results showed that SOC content (%) increased significantly in the upper 0–12 cm soil layer after *S. alterniflora* invaded the *Z. japonica* communities (Supplementary Table 1; *p* < 0.05). Due to the limitation of sampling methods, the difference in SOC density was unknown; and the difference in SOC in deep sediment (12–100 cm) at different regions should be investigated in the future.

In conclusion, we found that *S. alterniflora* pushed 14 m into the *Z. japonica* distribution region within 3 months and that the invasion ability of *S. alterniflora* was greater than the growth ability of *Z. japonica*. The growth of *Z. japonica* was inhibited gradually with the invasion of *S. alterniflora*. Our results describe the invasion pattern of *S. alterniflora* and can be used to develop strategies for the prevention and control of *S. alterniflora* invasion.

## Data Availability Statement

The raw data supporting the conclusions of this article will be made available by the authors, without undue reservation.

## Author Contributions

SY and YZ conceived the ideas and designed methodology and led the writing of the manuscript. SY, SCX, XZ, ML, YQ, RG, SX, and YuZ collected the data. SY analyzed the data. All authors contributed critically to the drafts and gave final approval for publication.

## Conflict of Interest

The authors declare that the research was conducted in the absence of any commercial or financial relationships that could be construed as a potential conflict of interest.

## Supplementary Material

Supplementary Table 1 The SOC content (%) at the three regions. Values are mean ± SD. Different letters indicate significant difference between different regions.

Supplementary Figure 1 *Spartina alterniflora* invasion of *Zostera japonica* meadows [**(A)** May 2015, low tide; **(B)** October 2016, high tide; **(C)** July 2019, low tide)].

Supplementary Figure 2 *Spartina alterniflora* invasion of *Zostera japonica* meadows in another site [**(A,B)** August 2015; **(C,D)** June 2020; the red five-pointed star was represented the location of this site]. ZJ, *Zostera japonica*; SA, *Spartina alterniflora*.

Supplementary Figure 3 Field photo of the study region [**(A)** April 27, 2019; **(B)** July 21, 2019].
