# Supplementary material for: Can the Non-native Salt Marsh Halophyte Spartina alterniflora Threaten Native Seagrass (Zostera japonica) Habitats? A Case Study in the Yellow River Delta, China
Source: Front Plant Sci. 2021 May 20;12:643425. doi: 10.3389/fpls.2021.643425 (PMC8173042; doi:10.3389/fpls.2021.643425)
Supplement: Supplementary Table 1 — The SOC content (%) at the three regions. Values are mean ± SD. Different letters indicate significant difference between different regions. [file Presentation_1.pdf]

Supplementary materials

Supplementary Table 1. The SOC content (%) at the three regions.

Region	SOC (%)
Spartina alterniflora	1.24 ± 0.16^{a}
Ecotone	1.14 ± 0.18^{a}
Zostera japonica	0.85 ± 0.04^{b}

Values are mean ± SD. Different letters indicate significant difference between different regions.

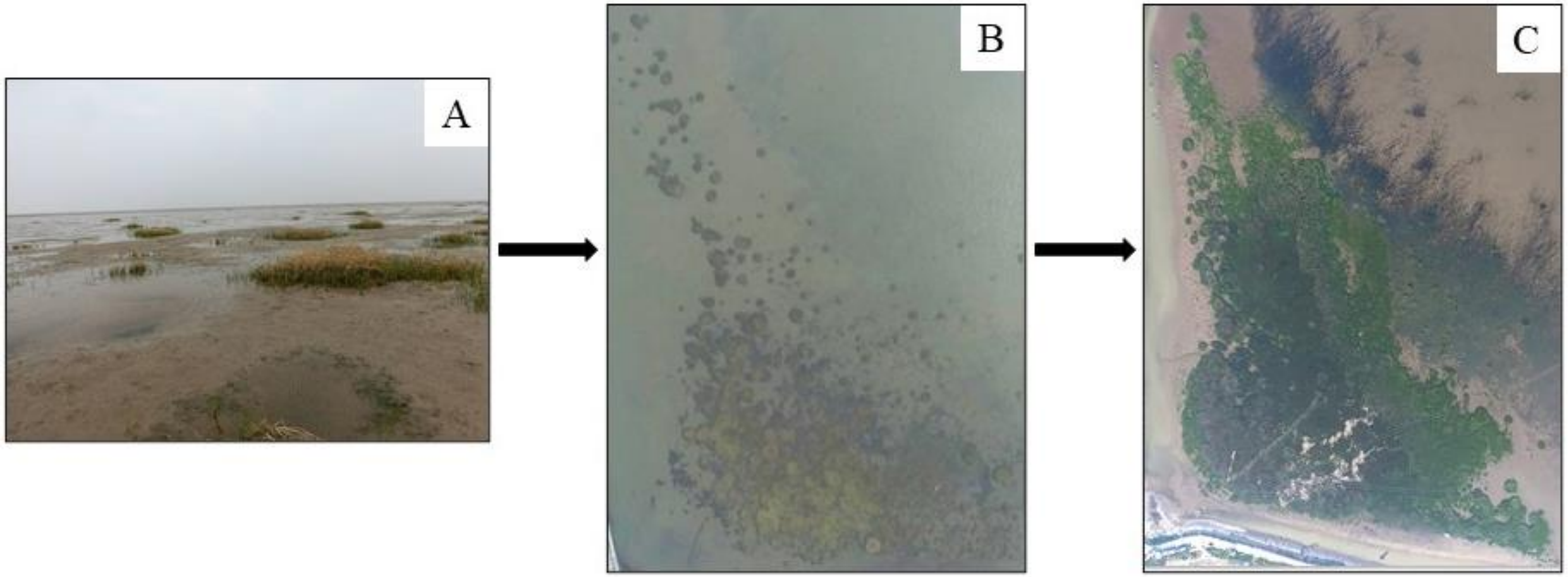

Supplementary Figure 1. *Spartina alterniflora* invasion of *Zostera japonica* meadows (A: May 2015, low tide; B: October 2016, high tide; C: July 2019, low tide).

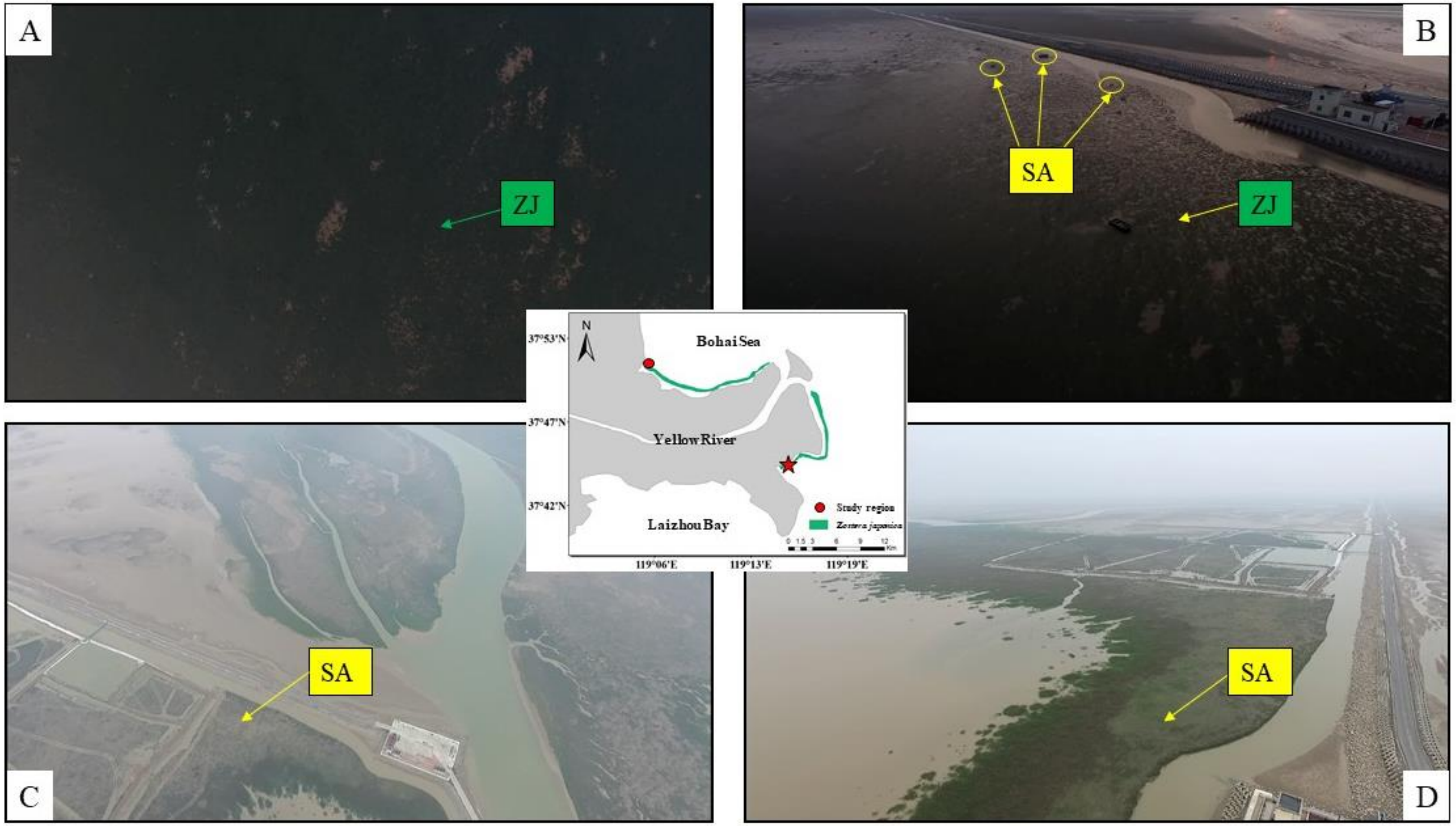

Supplementary Figure 2. *Spartina alterniflora* invasion of *Zostera japonica* meadows in another site (A, B: August 2015; C, D: June 2020; the red five-pointed star was represented the location of this site). ZJ, *Zostera japonica*; SA, *Spartina alterniflora*.

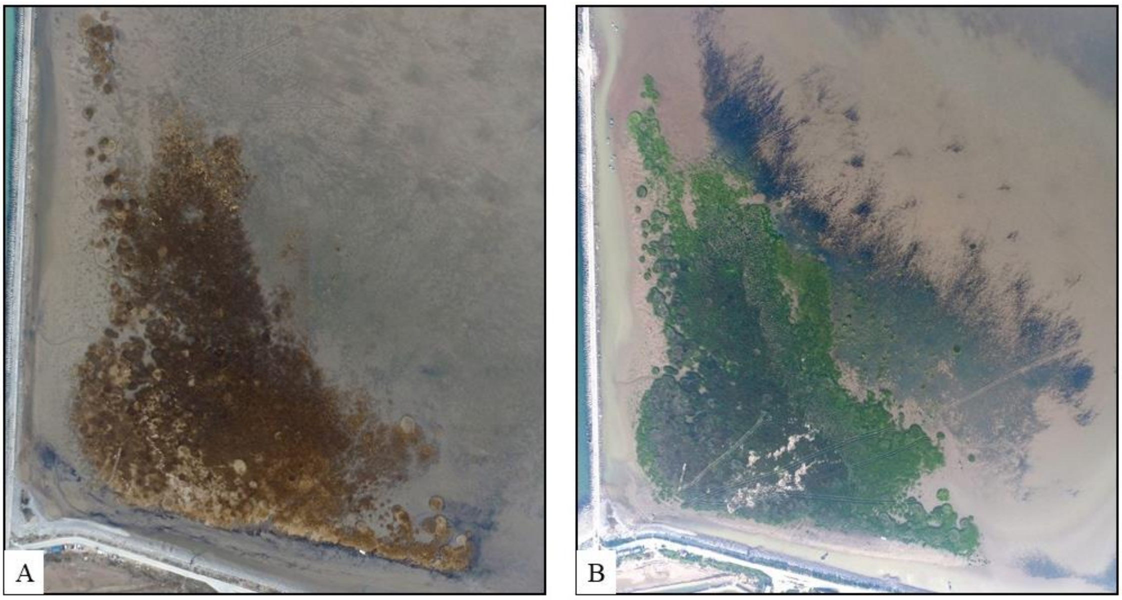

Supplementary Figure 3. Field photo of the study region (A: April 27, 2019; B: July 21, 2019).
